# Effect of Nanoparticles with Different Chemical Nature on the Stability and Rheology of Acrylamide Sodium Acrylate Copolymer/Chromium (III) Acetate Gel for Conformance Control Operations

**DOI:** 10.3390/nano10010074

**Published:** 2019-12-30

**Authors:** Saray Pérez-Robles, Cristian A. Matute, Jeison R. Lara, Sergio H. Lopera, Farid B. Cortés, Camilo A. Franco

**Affiliations:** 1 Grupo de Investigación en Fenómenos de Superficie-Michael Polanyi, Departamento de Procesos y Energía, Facultad de Minas, Universidad Nacional de Colombia, Sede Medellín, Medellín 050034, Colombia; sperezr@unal.edu.co (S.P.-R.); camatutem@unal.edu.co (C.A.M.); 2 Grupo de Investigación de Yacimientos de Hidrocarburos, Departamento de Procesos y Energía, Facultad de Minas, Universidad Nacional de Colombia, Sede Medellín, Medellín 050034, Colombia; jrlarad@unal.edu.co (J.R.L.); shlopera@unal.edu.co (S.H.L.)

**Keywords:** conformance, gel, EOR, nanotechnology, nanoparticles, rheology, syneresis, stability, viscoelasticity

## Abstract

During enhanced oil recovery (EOR), reservoir heterogeneities and fluids distributions promote preferential flow channels formation. Therefore, different types of gels have been proposed to improve swept efficiency on chemical flooding by plugging high permeability zones. The purpose of this article is to evaluate the effect that nanotechnology has on the inhibition of syneresis and the rheological properties of the Acrylamide Sodium Acrylate Copolymer/Chromium (III) Acetate gel system for conformance applications in mature reservoirs. Thus, a methodology is proposed in four stages: First, (I) nanoparticles synthesis, and characterization, followed by (II) bottle tests to monitor gelation kinetics and syneresis degree at 70 °C, then (III) description of the rheological evaluation on static and dynamic conditions to calculate gelation time and viscoelastic modulus (*G’* and *G”*), and finally (IV) the displacement test with the best gel system in the presence of nanoparticles. Results showed that the best nanoparticle was the chromium oxide (Cr_2_O_3_), which represented the lesser syneresis degree and increased gelation time. Syneresis of gel samples in the presence of Cr_2_O_3_ at day 30 was under 1% for gels prepared with 4000, 6000, and 8000 mg·L^−1^ of polymer, and polymer to crosslinker ratio (*p/c*) of 40:1. Regarding SiO_2_, MgO, and Al_2_O_3_ nanoparticles, results show an improvement of gel strength. However, their thermal stability in terms of syneresis was lower. Displacement test in a triple parallel Slim Tube was able to recover an additional 37% of oil of the total oil present in the sandpacks, confirming the effectivity of the system when 100 mg·L^−1^ of Cr_2_O_3_ nanoparticles are included.

## 1. Introduction

High permeability channels generate several problems in waterflooding processes due to the increment in water cut per oil barrel recovered and the incremental costs associated with water treatment [1]. Therefore, there are some practices such as conformance control to reduce poor sweep efficiency due to reservoir heterogeneities [2,3,4,5]. Conformance control treatments aim to recover unswept oil and gas trapped into low permeability paths looking forward to keeping a uniform profile of injected fluids across the productive zones in the reservoir and reduce water production [1,6,7]. Among the chemical methods used for this purpose, gels systems are one of the most applied around the world [8]. These types of systems are mainly composed of a high molecular weight polymer crosslinked with some organic or metallic agent and some additives, such as partially hydrolyzed polyacrylamide (HPAM) crosslinked with Cr^3+^ [9,10,11,12,13,14].

The stability of the gels system is subdued to the reservoir conditions such as temperature, water salinity, and pH, carbon dioxide (CO_2_) or hydrogen sulfide (H_2_S) presence, and adsorption of the reagents on the rock surface [15]. The latter can trigger a process known as syneresis consisting of water expulsion from the 3-dimensional network due to excessive chemical attractive forces within the gel structure or the lack of reagents to crosslink [16]. Gel degradation due to temperature conditions can be monitored through syneresis behavior and the main gel properties, gelation time and gel strength can be studied through rheological tests. In this way, several authors have studied the syneresis process in bottle tests and core samples for different systems. Nguyen et al. [17] measured the effect on gelation time while making variations in temperature, initial pH, polymer molecular weight, hydrolysis degree and concentration, polymer/crosslinker ratio, salt type, and concentration. The results showed that the gelation time decreases when temperature increases and that increasing polymer concentration or crosslinker concentration increases gelation time. Karimi et al. [12] studied syneresis behavior in an HPAM/Cr (III) polymer gel system under different parameters and realized syneresis increase with temperature and decrease with higher polymer concentrations.

Nanoparticles are proposed as a novel alternative to enhance polymer gel properties. In the past few years, nanotechnology has been widely studied in the oil and gas industry [18,19,20,21,22,23,24]. Nanoparticles are particles that present a high surface area to volume ratio and a diameter size between 1 nm and 100 nm [19]. Different chemical nature of the nanoparticles has been used, such as oxides of aluminum, zinc, zirconium, silicon, titanium, magnesium, iron, and many others modified in their surface facilizing the interactions between the reservoir phases [19,25,26,27].

Recently, Ma et al. [28] studied the gelation time, gel strength and thermal stability at 85 °C on the nano-silica hybrid HPAM/Polyethyleneimine (PEI) gel system and found that a mass fraction of 30% of colloidal silica nanoparticles maintained the gel stability for 27.5 days without syneresis. On the other hand, Dijvejin et al. [29] studied the effect of silica nanoparticles in concentrations between 2500 and 10,000 mg·L^−1^ on sulfonated polyacrylamide (SPAM)/Chromium and found an increase in the elastic and viscose moduli and, that by reducing the particle size and increasing concentration, the viscosity increase and the gelation is delayed.

However, to the best of our knowledge, there are no reports on the scientific literature about the use of low concentration (<100 mg·L^−1^) of nanoparticles of different chemical nature in the acrylamide/sodium acrylate copolymer/chromium (III) acetate gel system for improving gel stability by decreasing or inhibiting syneresis process. Hence, this study aims to evaluate the behavior of nanoparticles with different chemical nature (SiO_2_, Cr_2_O_3_, Al_2_O_3_, and MgO) when they are incorporated into an inorganically crosslinked gel system. Different aspects will be studied to establish whether nanoparticles improve gel characteristics such as syneresis percent, gelation time, and rheological properties (storage and loss modulus, *G’* and *G”*) at 70 °C. Different polymer concentrations were tested, while a polymer to crosslinker ratio (*p/c*) of 40:1 was kept constant. The displacement test in a triple parallel slim tube system confirmed the gel efficiency under dynamic conditions.

## 2. Materials and Methods

### 2.1. Materials

An acrylamide/sodium acrylate copolymer BG-100 (CAS N° 25987-30-8) in the form of white powder and a chromium acetate solution (11.1%, CAS N° 1066-30-4) as the crosslinker, were purchased from Flotek Industries, Inc. (Houston, TX, USA). The copolymer and the crosslinker were mixed in deionized water at different reagent dosages to perform this investigation.

The different nanoparticles used in this study were synthesized or purchased. The MgO and Cr_2_O_3_ nanoparticles were synthetized through the Sol-Gel and the electrochemical method, respectively. The commercial SiO_2_ and Al_2_O_3_ nanoparticles were purchased from Sigma Aldrich (St. Luis, MO, USA) and Petroraza S.A.S. (Sabaneta, Colombia), respectively. Full characterization and synthesis methods of Cr_2_O_3_, Al_2_O_3_, SiO_2_, and MgO nanoparticles can be found in our previous work [30], and some nanoparticle properties are summarized in Table 1.

### 2.2. Methods

#### 2.2.1. Gel Preparation

Gel samples with different polymer concentrations were prepared at room temperature using deionized water. First, a solution of 8000 mg·L^−1^ of the polymer was made, and dilutions to 6000, 4000, and 2000 mg·L^−1^ were obtained. After 24 h of additional stirring, the crosslinker was added drop by drop, keeping constant the polymer to crosslinker ratio in 40:1 for all concentrations. The nanoparticles were added to the mixture right after the crosslinker and mechanically stirred. Immediately, half of the volume of each gel sample was placed into sealed vials in an air forced oven at 70 °C for several days to follow syneresis development, and the other half was saved for the rheological test.

#### 2.2.2. Rheological Tests

A Kinexus Pro rotational rheometer (Malvern Instruments Ltd., Worcestershire, UK) was used to perform the rheological evaluation of the gel samples. The rheometer is equipped with both Peltier plate and cylinder cartridges for temperature setting to assure environmental control. All rheological tests will be conducted at 70 °C and atmospheric pressure.

Once the reagents are mixed, the polymer begins to create bonds with the crosslinker. In this sense, the gelation time is considered as the time that takes for the gelant solution to form a 3-dimensional network. The latter gives a precise idea of how long the gelant solution can flow through the porous medium reaching an adequate placement throughout the reservoir depth. The viscosity will be monitored as a function of time in a Couette geometry. A single shear rate (7.3 s^−1^) will be selected to compare the different viscosities at different polymer concentrations in the presence and absence of nanoparticles.

On the other hand, oscillatory measurements can provide qualitative information about the gel microstructure, such as solid-like behavior (storage modulus, *G’*) or liquid-like behavior (loss modulus, *G”*) [31]. Thus, a cone and plate geometry will be employed to obtain *G’*, and *G”*. Oscillation frequency range was taken from 1 to 100 Hz, and a strain of 5% was chosen after amplitude sweep test evaluations. The sample is placed in the lower plate, and a movable 4°/45 mm cone is then placed automatically on top of it.

All rheological measurements were performed by triplicate with deviations lower than 3%.

#### 2.2.3. Syneresis Measurements

Syneresis percent was measured using the weighting method [12]. As soon as syneresis began, two phases could be observed in the sealed vials. Expelled water settled on top of the remaining gel. Each gel sample was put out of the oven, then water was pulled out with a syringe, and the gel sample was weighted using an analytic balance. The samples were sealed and put back into the air forced oven at 70 °C.

Syneresis estimation was made comparing the initial mass of the gelant with the remaining mass of the gel every time water was removed. The syneresis percent ( %Syn) calculation was made with the following equation:

(1) $$\%Syn=\left(\frac{m_{1}-m_{2}}{m_{i}}\right)100\%$$

where, $m_{1}$ is the gel weight placed in the vial, $m_{2}$ is the weight of the vial once the water has been pulled out of it and $m_{i}$ is the weight of gel, dismissing the weight of the vial.

The syneresis tracking of the samples was realized by triplicate with deviations lower than 5%.

#### 2.2.4. Sydansk’s Code

Gelation kinetics can also be monitored through Sydansk’s code, which consists of a visual evaluation of the gel behavior upon the sealed vial inversion. A scale from 1 to 10 describes the different stages of gelation performance qualitatively as a time function. The description of the bottle test is based on Sydansk’s Code 1988 [32], and results are summarized in Table 2. The small letters describe an arbitrary gel elasticity, i.e., the *n*, *s*, *g*, and *e* are abbreviations for no sign of gelling, slight tendency to gel, good gel elasticity, and excellent gel elasticity, respectively. Each bottle is examined in the hour and daily while the samples are inverted, with everyday data a description table is constructed [3].

#### 2.2.5. Displacement Test in a Triple Parallel Slim Tube System

When EOR processes such as waterflooding are applied in mature heterogeneous reservoirs fingering, and water channeling often occurs decreasing sweep efficiency. A simulation of a heterogeneous porous media can be realized in triple parallel assembly by using different sizes of sand to obtain different permeabilities. The evaluation of a plugging gel for conformance control can be realized in this system to analyze the additional oil recovery and the conductivity of the porous medium. Therefore, waterflooding before and after the implementation of a gel to block the swept channels was performed. The equipment used consist of three Slim Tubes disposed in parallel and filled with Ottawa sand mesh sizes 8–20 (17%), 40–70 (50%), and 100–200 (33%) for the first sand pack, 20–40 (100%) for the second pack, and 8–20 (10%), 40–70 (60%) and 100–200 (30%) for the third one. The fluids used in the tests were a 7000 mg·L^−1^ of NaCl as brine and a light crude oil of 34° API.

**Figure 1:**
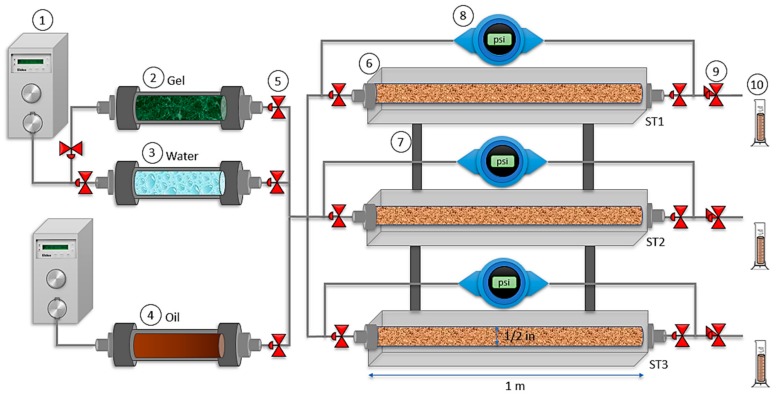
Experimental assembly of a parallel triple slim tube system: (**1**) Injection pump, (**2**) gel displacement cylinder, (**3**) water displacement cylinder, (**4**) oil displacement cylinder, (**5**) control valve, (**6**) slim tube, (**7**) support bar (**8**) pressure transducer, (**9**) back pressure valve, and (**10**) fluid collector. The dimensions of the slim tubes are 1 m of length and 1.27 cm of inner diameter.

The triple Slim Tube assembly (Figure 1) consists of three Slim Tubes of 1 m of length and a half-inch of inner diameter. A heating system covers each slim tube for temperature control. A low flow piston pump and a positive displacement cylinder were used to inject the fluids into the porous medium. Rosemount 3051 (Emerson Electric Co., St. Louis, MO, USA), differential pressure transducers, were used to monitor the pressure in each sand pack. The total pore volume (PV) and absolute permeability (*K_abs_*) were calculated by filling each slim tube with brine. Then, each porous medium was separately saturated with crude oil until irreducible water saturation (*S_wir_*) condition. At this point, each Slim Tube oil conductivity was measure considering their differential pressure at constant fluid flow rate injection. Then, a conventional waterflooding was made at the same time in the three sand packs, i.e., in parallel until irreducible oil saturation (*S_or_*). After that, the gel was also injected in parallel into the system at room temperature to avoid premature gelation. Furthermore, a specific time to assure gelation was kept before the second waterflooding according to the rheological measurements. A flow injection rate of 0.3 mL/min was used to inject all fluids, and a volume of gel equal to the produced oil from the high permeability slim tube was injected to block the preferential channel and recover additional oil from the other sand packs. All experiments were carried out at 70 °C and a 13.8 MPa of overburden pressure.

## 3. Results and Discussion

### 3.1. Materials Characterization

**Figure 2:**
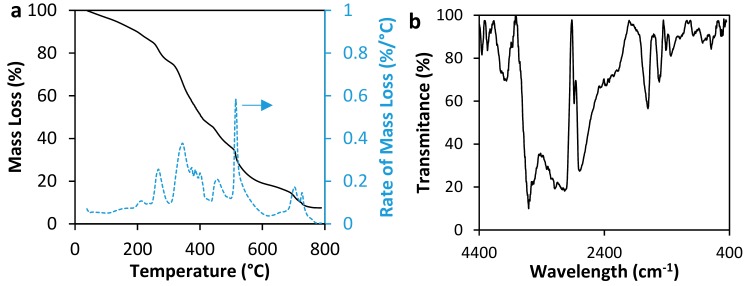
Characterization of acrylamide/sodium acrylate copolymer by (**a**) TGA and (**b**) FTIR techniques.

Figure 2 shows the results of polymer characterization by thermogravimetric analyses (TGA) and Fourier Transformed Infrared Spectroscopy (FTIR) techniques. Regarding FTIR analysis, the band observed around 1010 cm^−1^–1110 cm^−1^ is related to C-C stretching vibration, the bands at 1380 cm^−1^ and 1580 cm^−1^ shows the presence of N-H bonds related to (-NH_2_) and (-CONH_2_) groups [33,34]. The bands at 1410 cm^−1^ and 1560 cm^−1^ are related to COO^−^ of Na-acrylate [35,36], and the band at 1700 cm^−1^ shows the C=O bonds [33,35]. At 2967 cm^−1^, there is a band related to symmetry and asymmetry to C-H bonds [37]. Bands between 3170 cm^−1^–3356 cm^−1^ are related to moisture present in the polymer [33,34]. The bands between 3000 cm^−1^–3500 cm^−1^ are characteristics of (-OH) bonds present in carboxylic groups (-COOH) [33,34]. There are some differences (shape, position, and amplitude of bands) among conventional polymers’ FTIR spectra and the acrylamide sodium acrylate polymer’s spectra. This is attributed to changes relating to interactions among Na^+^ and the polymer chain [38].

On the other hand, results from TGA present significant deterioration of the polymer at 270 °C. At this point, almost 20% of the mass has been lost. For this study, it should be considered that the mass lost before 100 °C is nearly 3%, which could correspond to humidity lost. In general, the decomposition rate shows three principal decomposition peaks at 270 °C, 350 °C and 515 °C.

### 3.2. Rheological Measurements

#### 3.2.1. Gelation Time

**Figure 3:**
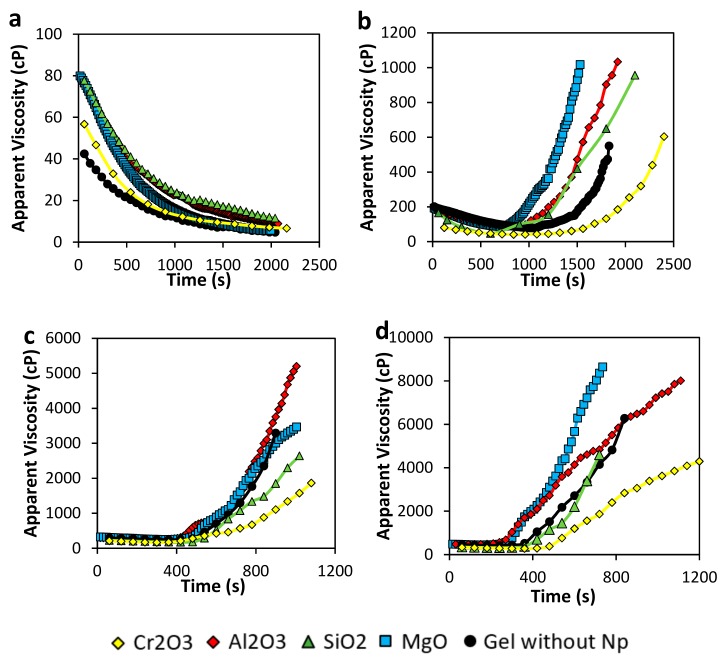
Gelation time of gel samples in the presence and absence of nanoparticles at (**a**) 2000 mg·L^−1^, (**b**) 4000 mg·L^−1^, (**c**) 6000 mg·L^−1^, and (**d**) 8000 mg·L^−1^ of polymer concentration and (**a**) polymer to crosslinker ratio of 40:1 at 70 °C.

Results of gelation time estimated as the inflection point in the curve of apparent viscosity vs. time are shown in Figure 3. In Figure 3a, using a 2000 mg·L^−1^ of Acrylamide Sodium Acrylate Copolymer concentration to form the gel, viscosity only decreases with time. Initial sample gel in the absence of nanoparticles viscosity is around 40 cP, and as time goes by the viscosity decrease to values lesser than 10 cP. All nanoparticles tend to increase initial viscosity at even the double of the system without nanoparticles (from 40 to 80 cP). However, the viscosity decreases to values of less than 15 cP. Therefore, as no inflection point is recorded, no gelation is completed nor in the presence or absence of any of the different nanoparticles. The low polymer concentration, combined with the increasing of temperature in the sample, results in poor crosslinking reactions.

Regarding the samples prepared with 4000 mg·L^−1^ of the polymer showed in Figure 3b, viscosity initially decreases due to temperature. However, the inflection point begins between 750 and 1500 s, confirming the formation of a tridimensional network at different times, depending on the nature of the nanoparticle added. In this case, gelation time is shorter respect to the sample without nanoparticles when MgO nanoparticles are included in the gel, followed by the SiO_2_ and Al_2_O_3_ nanoparticles, with gelation times around 750, 900, and 1000 s, respectively. This result could be explained due to these nanoparticles crosslinking effect in the gel, accelerating the bonding creation. However, storage modulus and Sydansk’s code showed no gel formation for the gel in the absence of nanoparticles; a slight increasing in viscosity was observed for this sample. A constant shear rate of 7.3 s^−1^ applied to the gel to perform the analysis could promote the formation of a weak 3-dimentional network. On the other hand, Cr_2_O_3_ nanoparticles delay crosslinking reaction which implicates that this sample could reach a longer in-deep location if used in a conformance control operation.

Results showed in Figure 3c represents the gelation time for samples prepared with 6000 mg·L^−1^ of the polymer. Comparing with the previous results in 4000 mg·L^−1^, if polymer concentration increase, gelation time decrease, as it is logical due to a higher amount of carboxylic group and crosslinker available to interact and create ionic bonds, as was described by Nguyen et al. and Karimi et al. [12,17]. In samples presented here, they all approximately begin to become a rigid gel at 8 min, and after that, it takes another 8 min to increase their viscosity to the maximum. The difference respect to the static gelation time calculation by Sydansk’s code, which takes approximately 2 h to start gelation, is that sample heating occurs faster due to the cylinder geometry rotation, which also promotes reagents interactions. Gelation time occurs very similar to the previous composition in b. It follows the sequence Cr_2_O_3_ > SiO_2_ > Gel without nanoparticles > MgO > Al_2_O_3_.

Finally, Figure 3d presents the results of gelation time corresponding to the samples prepared with 8000 mg·L^−1^ of the polymer. We can observe gelation time occurs as follow: Cr_2_O_3_ > SiO_2_ > Gel without nanoparticles > Al_2_O_3_ > MgO nanoparticles. Also, as the gelation starts faster than lower concentrations of polymer, the speed of viscosity rising is higher (higher slope), and the maximum viscosity value could reach more than 8000 cP.

#### 3.2.2. Viscoelastic Modulus (G’ and G”)

The storage modulus (*G’*) is the measuring of the amount of energy that structured liquids require when deformation is applied to the fluid, and the minimum-energy state is altered, making thermodynamic forces activate to return to its natural rest condition [31,39]. In other words, structured fluids in rest condition, storage minimum energy that will always be the target condition when elastic forces try to return to the original state. On its behalf, loss modulus (*G”*) represents the viscous response of the fluid and quantifies the amount of mechanical energy that dissipates as heat and movement due to the elastic forces that operate inside the liquid system [11].

The gel samples respond to angular frequency (ω, rad·s^−1^) under a constant oscillatory strain force. *G’* and *G”* are analyzed considering polymer concentrations from 2000 to 8000 mg·L^−1^ and the different nanoparticles’ nature (SiO_2_, MgO, Al_2_O_3_, and Cr_2_O_3_), and a *p/c* ratio of 40:1. At higher frequencies (short times), the behavior of the samples is dominated by an elastic response, and the viscous response governs in the low frequencies [31]. For all samples, *G’* increases with increasing the angular frequency in the range of 1–100 ω. In this way, the gel strength given by the amount of energy that the network is capable of storage is bigger when the strain is applied in short times.

**Figure 4:**
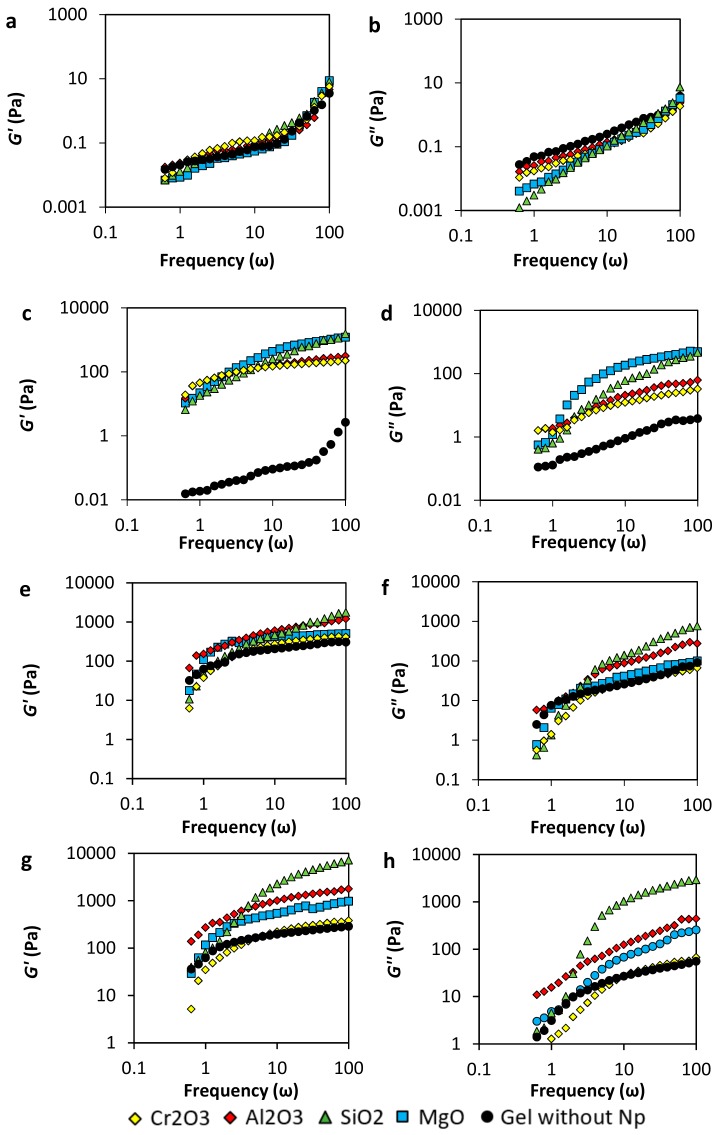
Storage moduli (*G’*) and loss moduli (*G”*) of acrylamide/sodium acrylate copolymer/chromium III acetate gel system at a fixed dosage of 100 mg·L^-1^ of Al_2_O_3_, MgO, Cr_2_O_3_, SiO_2_ nanoparticles, a polymer concentration of (**a**,**b**) 2000 mg·L^−1^, (**c**,**d**) 4000 mg·L^−1^, (**e**,**f**) 6000 mg·L^−1^, and (**g**,**h**) 8000 mg·L^−1^ and 70 °C.

In Figure 4 (panels a and b), *G’* and *G”* of samples prepared with 2000 mg·L^−1^ of polymer are shown. The magnitude of *G”* is slightly bigger than *G’* for the sample without nanoparticles in the range of frequency evaluated, which means that the viscous behavior predominated over the solid one. This result indicates that copolymer chains were not enough to crosslink with the chromium acetate, and no gel network was completely formed. On the other hand, in the presence of nanoparticles, the viscous response is lower in the presence of nanoparticles, which means that the nanoparticles decrease the viscosity of the samples. The low nanoparticle concentration could lead to the interaction with the polymer and the crosslinker, depending on the surface charge, forming nucleation points that decrease the entanglement.

In Figure 4 (panels c and d) are presented the results for gel samples prepared with a polymer concentration of 4000 mg·L^−1^. The sample without nanoparticles shows that the viscous behavior predominates, indicating that no tridimensional network was produced. Comparing the *G’* and *G”* moduli, it is notorious that there is an improving in the gel structure due to the presence of nanoparticles (Figure 4c). MgO and SiO_2_ nanoparticles show a higher increase of *G’* compared with Al_2_O_3_ and Cr_2_O_3_ nanoparticles. However, as it can be observed from Figure 4d, the last ones dissipate the energy in less quantity than the other nanoparticles (lower *G”*). In this sense, a higher amount of the energy applied to deform the Al_2_O_3_ and Cr_2_O_3_ gels is recovered, and more energy is lost with the MgO and SiO_2_ gels. The addition of nanoparticles can be used for the decreasing of the polymer concentration in the gel elaboration, obtaining similar characteristics, which would be a very significant improvement in the cost to benefit ratio in a field application.

Results of viscoelastic modulus for gel samples prepared with 6000 mg·L^−1^ of the polymer are presented in Figure 4 (panels e and f). The system Acrylamide Sodium Acrylate Copolymer /Chromium III Acetate in the absence of nanoparticles reaches gel formation showing *G’* values bigger than *G”*, confirming the 3-dimensional network formation. It is worth noticing that the *G’* values of this gel are similar to those of gels prepared with lower polymer concentration (4000 mg∙L^−1^ in panel c) in the presence of nanoparticles, confirming the possibility to decrease polymer concentration obtaining similar gel characteristics.

Also, results obtained for gels with 8000 mg·L^−1^ of the polymer are presented in panels g and h. In general, comparing both Figure 4g,h, it is observed that the *G’* is larger than the *G”* and that the elastic modulus increases with the addition of the nanoparticles, strengthening the 3-dimensional network. However, as *G’* increases, *G”* increases, except for the sample with Cr_2_O_3_ nanoparticles in which the elastic modulus does not increase, and the *G”* decreases at frequencies below 10 ω.

**Figure 5:**
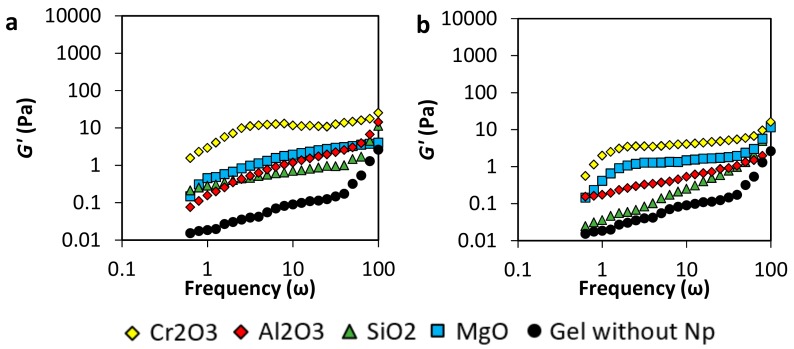
Storage moduli of acrylamide/sodium acrylate copolymer /chromium III acetate gel system at dosages of (**a**) 50 mg·L^−1^ and (**b**) 20 mg·L^−1^ of Al_2_O_3_, MgO, Cr_2_O_3_, SiO_2_ nanoparticles, a polymer concentration of 4000 mg·L^−1^, and 70 °C.

On the other hand, the evaluation of the nanoparticles’ concentration effect was evaluated. Results in Figure 5 show the storage moduli (*G’*) of the gels samples of 4000 mg·L^−1^ of the polymer at low nanoparticles concentration, i.e., 50 mg·L^−1^ and 20 mg·L^−1^. The decreasing of the concentration harms gel formation. This result could be explained if the nanoparticles are considered as nucleation points that have a similar roll to the crosslinker agent. Therefore, the reduction of the concentration of the nanomaterials decreases the value of *G’*, forming weaker gels to almost showing no gel at all. However, Cr_2_O_3_ nanoparticles show the mayor improving the effect, followed by the MgO nanoparticles and the Al_2_O_3_, and finally by the SiO_2_ nanoparticles.

### 3.3. Syneresis Behavior

Syneresis process is a thermodynamic equilibrium phenomenon that results in the shrinking of the gel network due to the excessive chemical attractive forces between the polymer and the crosslinker, that conduct to the expulsion of water from an aqueous gel [40]. The addition of crosslinker in a disproportionate way regarding the polymer is one of the principal reasons that lead to syneresis. A crosslinker concentration above the optimal level produces too many bonds, and below creates poor gel strength. The excess crosslinking active sites can come from the intentional addition of the chemical to the gel formula or due to polymer auto hydrolysis [40].

Monitoring the syneresis development was made using a syringe to extract the water from the samples when the gel samples were taken out of the oven. Results showed in this section are about syneresis developed for gel samples prepared with 4000, 6000, and 8000 mg·L^−1^ of polymer concentration when subduing to 70 °C. In the experiments, a 40:1 of *p/c* ratio was kept constant, and 100 mg·L^−1^ of concentration of SiO_2_, Al_2_O_3_, MgO, and Cr_2_O_3_ nanoparticles were added to each vial. Regarding 2000 mg·L^−1^ acrylamide/sodium acrylate copolymer/chromium (III) acetate samples, no gel formation was achieved at the test conditions.

**Figure 6:**
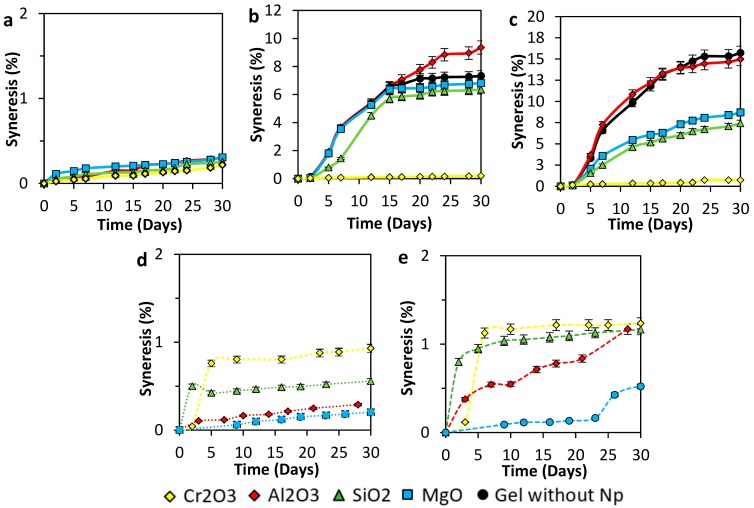
Syneresis development of (**a**) 4000 mg·L^−1^, (**b**) 6000 mg·L^−1^, and (**c**) 8000 mg·L^−1^ of polymer concentration of acrylamide sodium acrylate copolymer/chromium III acetate gel systems at a fixed dosage of 100 mg·L^−1^ of Al_2_O_3_, MgO, Cr_2_O_3_, and SiO_2_ nanoparticles, as well as for a polymer concentration of (**a**) 4000 mg·L^−1^ and different nanoparticles concentrations of (**d**) 50 mg·L^−1^ and (**e**) 20 mg·L^−1^. Tests were performed at a polymer to crosslinker ratio of 40:1 and 70 °C.

Figure 6a shows the syneresis of 4000 mg·L^−1^ of polymer concentration gel samples in the presence of nanoparticles. The control sample did not form a gel, so the syneresis could not be registered. The low polymer concentration and the test temperature promote polymer degradation instead of crosslinking interactions. On the other hand, all the samples with nanoparticles favored gel formation. The formulation with Cr_2_O_3_ nanoparticles showed the best syneresis inhibition with 0.18% of water loss in 30 days, followed by formulation with SiO_2_ nanoparticles that shows 0.24% of syneresis at the same time. Formulation with MgO and Al_2_O_3_ nanoparticles show similar syneresis performance with approximately 0.28% of expelled water.

Figure 6b shows the syneresis effect for gel samples with 6000 mg·L^−1^ of polymer concentration, polymer to cross-linker ratio 40:1, and 100 mg·L^−1^ of different nanoparticles concentration. With these concentration all the samples formed a gel, base sample had a maximum syneresis of 7%, in comparison with the results of formulations that include nanoparticles we found that formulation with Cr_2_O_3_ nanoparticles exhibit the best syneresis performance again but this time with a water loss of 0.2% in 30 days of test, followed by formulation with silica nanoparticles that show 6.4% of maximum water loss in the same time of experiment similar to formulation with magnesium that display 6% of maximum syneresis. Alumina nanoparticles present a poor performance with a water loss of 9%, which is 2% more than the base sample.

Syneresis development progress of gel simples made with 8000 mg·L^−1^ of polymer with *p/c* ratio of 40:1 are presented in Figure 6c. Gel without nanoparticles reaches a syneresis percent of 16% at day 30, followed closely by Al_2_O_3_ gel with 15% at the same time due to an excess of crosslinking forces. On the other hand, MgO and SiO_2_ nanoparticles reduce syneresis degree to 9 and 7.5%, respectively. However, these nanoparticles enhance the gel stability at temperature conditions, the Cr_2_O_3_ nanoparticles present and outstanding behavior in the inhibition of the network degradation. The Cr_2_O_3_ nanoparticles prevent syneresis in 93.75% during the first 30 days.

On the other hand, Figure 6d,e represent the results of the syneresis behavior of gels performed at lower nanoparticles concentration (20 and 50 mg·L^−1^). It is possible to infer from Figure 6d that the nanoparticles inhibit the gel degradation at least 87.5%. However, decreasing the concentration of nanoparticles even more (Figure 6e), increases syneresis, showing a predominance of the degradation process over the crosslinking.

### 3.4. Gelation Kineticks by Sydansk’s Code

The Sydansk’s code test was carried out at 2 h, 4 h, 8 h, 24 h, 48 h, one week, two weeks, three weeks, and four weeks after the gel’s formation. Table 3 shows the results of the Sydansk’s code in the presence of nanoparticles. First, gel without nanoparticles was evaluated to have a reference system to determinate nanoparticles influence in gel strength as a function of time.

For 2000 mg·L^−1^ of polymer-based gels, no visual sign of gelation was detected for any sample during the test, including the samples with nanoparticles. These results agree with the findings in the rheological studies of storage modulus and apparent viscosity. It could be explained due to the low polymer concentration and the relatively high temperature (70 °C) of the test, which allow degradation effect to be higher than the interaction forces between the Cr^3+^ of the crosslinker and the carboxylic group present in the acrylamide/sodium acrylate copolymer.

Comparing the gels prepared with 4000 mg·L^−1^ of polymer in the presence and absence of nanoparticles, we can observe that all materials enhance gel behavior from scale 1 to 3 in Sydansk’s code, which indicates that a viscoelastic network is formed in the presence of nanoparticles while no gel is observed in the absence of them at 70 °C. These results agree with those obtained in the rheological test. Regarding the gels formed with 6000 mg·L^−1^ of polymer, different effects can be seen for the different nanoparticles. For the control gel, the final gel strength of 6 in the scale was reached with good gel elasticity. Meanwhile, for the gel samples with Al_2_O_3_, a notorious improvement in thermal stability and gel strength was observed. In this sample, a semi rigid gel which surface barely got disturbed upon inversion was obtained (9 in the scale). The MgO nanoparticles’ effect was also good; the gel increased its viscoelasticity obtaining a value of 8 in the Sydansk’s code. This means that the surface of the gel got a high deformation upon inversion, but it reaches less than half of the bottle. On the other hand, Cr_2_O_3_ and SiO_2_ nanoparticles slightly improve gel performance respect to the reference sample. A value of 7 in the Sydansk’s scale at day 30 was obtained for both gels.

Finally, the 8000 mg·L^−1^ polymer concentration gel, the same previous tendency, was followed. The control gel was characterized with a value of 7 in the scale. Al_2_O_3_ and MgO nanoparticles resulted in an excellent gel elasticity (10 in Sydansk’s code) while SiO_2_ and Cr_2_O_3_ enhanced the gel to a lesser degree (9 and 8, respectively). These results could explain the syneresis behavior due to an excess of intramolecular forces between the polymer and the crosslinkers, in this case, nanoparticles of SiO_2_, Al_2_O_3_ and MgO present more syneresis while Cr_2_O_3_ that create weaker gel respect to the other nanoparticles develop a significantly lower syneresis degree.

Considering the gel pH ~ 5 and the nanoparticles Zeta Potential, the results could obey to a sequence. Al_2_O_3_ and MgO nanoparticles have positive superficial charges at the gel pH, promoting nucleation points where the carboxylic group of the polymer can form an entanglement. Regarding, SiO_2_ nanoparticles, they present a negative zeta potential at the work pH ~ 5, which results in weaker gel strength. Interactions between the chromium acetate and the SiO_2_ nanoparticles are most likely to occur due to their opposite charges. Finally, Cr_2_O_3_ nanoparticles have their point of cero charge at exactly pH ~ 5, which means that they have the same amount of positive and negative surface charges.

### 3.5. Syneresys Prediction through Nanoparticles Zeta Potential

**Figure 7:**
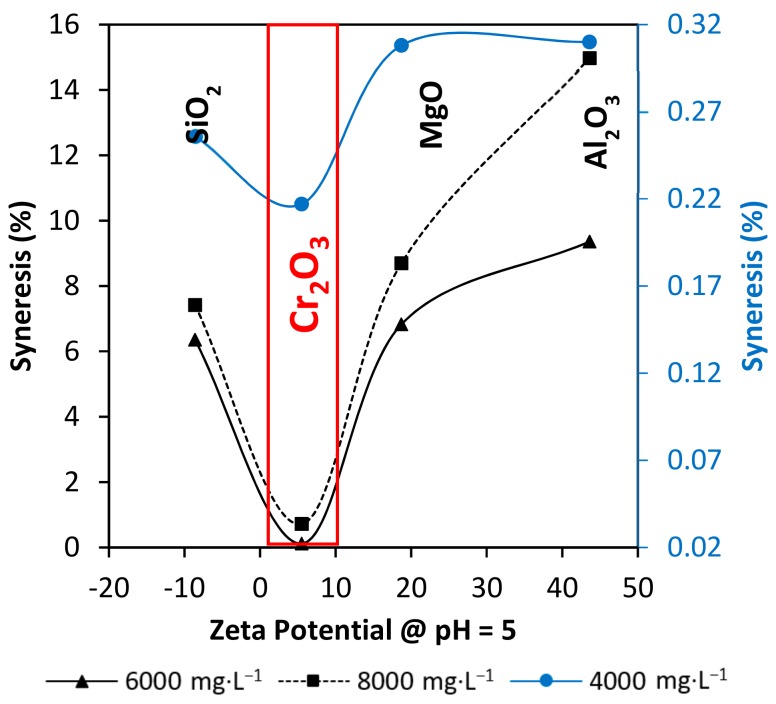
Correlation between nanoparticles Zeta Potential at the pH of work and syneresis development at 70 °C on day 30.

Figure 7 represents the nanoparticles Zeta Potential and the syneresis development relation at the pH of work. The superficial charges of the nanoparticles at pH = 5 is a key parameter to determinate their interaction with the polymer and the crosslinker in the gel. At the test conditions, the closer to the neutral charge states in the nanoparticles surface, the better. In this way, Cr_2_O_3_ nanoparticles offer the best scenario to interact with the polymer carboxyl group and the chromium cation (Cr^3+^). Al_2_O_3_ and MgO nanoparticles charged with positive charges may hinder polymer and crosslinker interactions by polymer adsorption without creating a 3-dimensional network. The SiO_2_ nanoparticles perform similar behavior with negative charges at pH = 5. SiO_2_ nanoparticles may be repelled with the carboxyl group of the polymer, leading to a weak gel behavior.

### 3.6. Displacement Test Results

Results of the evaluation of the gel with 4000 mg·L^−1^ of polymer and a *p/c* of 40:1 in the presence of 100 mg·L^−1^ of Cr_2_O_3_ nanoparticles are presented in this section. Results of the absolute permeabilities of the slim tubes used in the test report different values. For the first slim tube, 73 mD of permeability was obtained (low permeability), followed by the second slim tube the value was 1341 mD of permeability (high permeability), and finally, the third slim tube with 128 mD (medium permeability).

**Figure 8:**
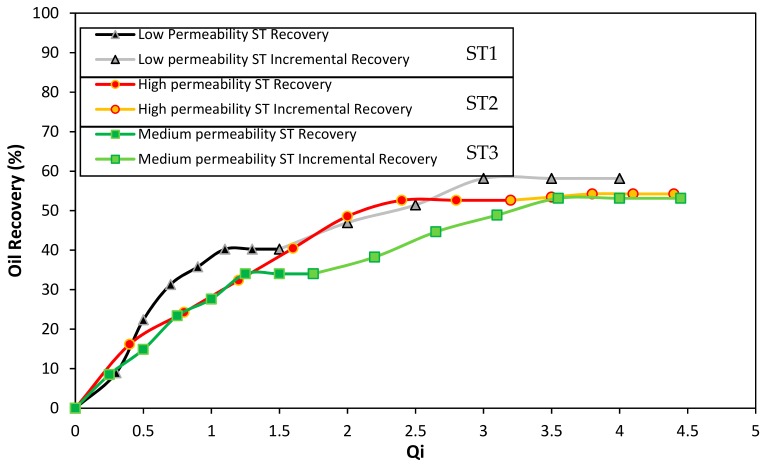
Oil recovery through waterflooding before and after the injection of the best gel obtained. The porous medium is composed of Ottawa sand mesh sizes 8–20 (17%), 40–70 (50%), and 100–200 (33%) for the low permeability slim tube (ST1), 20–40 (100%) for the high permeability sand pack (ST2), and 8–20 (10%), 40–70 (60%), and 100–200 (30%) for the medium permeability slim tube (ST3). Waterflooding before and after the gel injection was made with a 7000 mg/L of NaCl brine. The gel systems consisted of 4000 mg·L^−1^ of Acrylamide Sodium Acrylate/Chromium (III) Acetate in *p/c* ratio of 40:1 in the presence of 100 mg·L^−1^ of Cr_2_O_3_ nanoparticles. The test was performed at 60 °C and 13.8 MPa of overburden pressure.

Figure 8 shows the oil recovery as a function of the total system injectivity (Qi). In the triple parallel Slim Tube system, it is difficult to certainty know the pore volumes injected in each sandpack when the injection is made in parallel. Therefore, the total cumulated volume of oil produced from the system is normalized respect to the total cumulated volumes of brine injected. At the initial recovery in the system, as expected, channeling through the high permeability slim tube (ST2) occurred. Therefore, oil recovery reached 52% of the total oil in the sand pack. This value is very high compared to 40% and 34% of the low and medium permeability slim tubes (ST1 and ST3), respectively. Also, at 1.5 Qi, the channeling in these slim tubes reached the maximum amount of oil recovered, indicating that the swept efficiency was very low compared to the high permeability slim tube results, in which the maximum oil recovery was obtained after a value of 3 of Qi.

**Figure 9:**
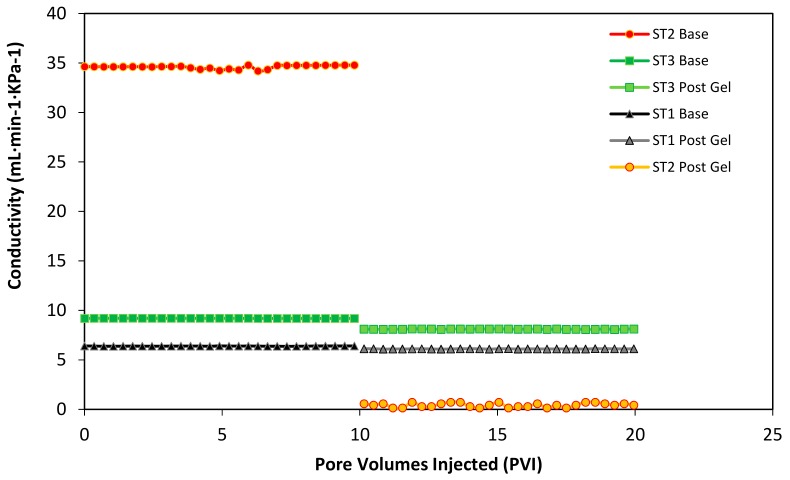
The conductivity of the porous medium before and after gel injection as a function of the pore volumes injected (PVI) of oil in each Slim Tube separately. Base curves correspond to the conductivity before the first waterflooding and post gel curves to the reduced conductivity after the gel plugging. The porous medium is composed of Ottawa sand mesh sizes 8–20 (17%), 40–70 (50%), and 100–200 (33%) for the low permeability slim tube (ST1), 20–40 (100%) for the high permeability sand pack (ST2), and 8–20 (10%), 40–70 (60%) and 100–200 (30%) for the medium permeability slim tube (ST3. The injection rate was kept constant at 5 mL·min^−1^. The test was performed at 60 °C and 13.8 MPa of overburden pressure.

After the initial recovery, the injection of the gel was made in parallel, i.e., all the three control valves that enter the slim tubes (ST1, ST2, and ST3) were open as the gel of the displacement cylinder was pumped. To assure gelation time, the system was closed 12 h before restarting the waterflooding. In this stage, the higher amount of gel was positioned in the most permeable slim tube due to the low resistance to flow and the high permeability. The above can be confirmed by the analysis of the system conductivity in Figure 9 that will be provided further. Regarding the incremental oil recovered from the system at 4 Qi, a 58% recovery factor was obtained for the ST1, while 53% was the result for the ST2 and ST3. In this sense, increases respect to the initial recovery of 55.9% and 45% were obtained for the medium and low permeability Slim Tubes. It is worth to mention that the low incremental oil recovery from the high permeability channel (ST2) may be due to the high percent of recovery reached in the initial waterflooding.

Additionally, each Slim Tube conductivity was monitored before the initial waterflooding and after the waterflooding after the gel injection. Initially, when the slim tubes are individually saturated with oil at the irreducible water saturation, it is possible to measure the differential pressure of the sand pack at a constant flow injection rate condition. This process allows building a conductivity curve for each slim tube as a function of the pore volumes injected (PVI). In Figure 9, the first 10 PVI corresponds to the conductivity of the different permeability sand packs before the gel injection. It is evident that the higher conductivity corresponds to the second Slim Tube (ST2), with an average of 34.519 mL·min^−1^·KPa^−1^, followed by the third Slim Tube (ST3) with 9.282 mL·min^−1^·KPa^−1^, and by last the first Slim Tube (ST1) with 6.382 mL·min^−1^·KPa^−1^ approximately. Then, after the gel injection at the end of the second waterflooding, each sand pack was individually refilled with oil to evaluate the conductivity changes produced by the blocking agent. Several pore volumes were injected to try to reach the initial oil saturation. However, the conductivity of each system decreased inversely proportional to the value of its permeability. In this sense, the second Slim Tube (ST2) lost almost 100% of is conductivity, followed by the third Slim Tube (ST3) with 12.5% and 4.5% of the first Slim Tube (ST1). These results, resumed in Table 4 indicate that the higher amount of plugging gel was successfully located in the high permeability Slim Tube, while the low quantity of gel entered the medium and low permeabilities Slim Tubes (ST3 and ST1).

## 4. Conclusions

Acrylamide/sodium acrylate copolymer crosslinked with inorganic chromium (III) acetate with the inclusion of different low concentration of nanoparticles (100 mg∙L^−1^) were developed. Gelation kinetic and gel strength were monitored by rheological measurements and Sydansk’s Code, while thermal stability was followed by syneresis behavior. Nanoparticles and polymer were physicochemically characterized. Regarding gelation time, Cr_2_O_3_ nanoparticles tend to delay gel formation which is favorable for gel location into the target zone. However, Al_2_O_3_, MgO, and SiO_2_ accelerate the gelation process. Viscoelastic modulus results also showed an improvement in gel strength when nanoparticles are added to the gel system, especially when the polymer concentration is 4000 mg∙L^−1^. In this case, solid-like behavior predominates over de liquid-like due to the nanoparticles’ effect, i.e., no gel formation is detected when the gel is only made with polymer and crosslinker, which could lead to decrease polymer concentration. Therefore, a reduction in the polymer concentration can be made if nanoparticles are added to the system, obtaining an effective gel behavior. Moreover, thermal stability results showed that Cr_2_O_3_ nanoparticles enhance gel perdurability when it is subjected to 70 °C. At 4000 mg∙L^−1^ of polymer, gels with nanoparticles developed syneresis degree lees than 0.4% in 30 days while for 6000 mg∙L^−1^ of polymer, syneresis result follows the order Al_2_O_3_ < Gel without nanoparticles < MgO < SiO_2_ < Cr_2_O_3_, meaning that Al_2_O_3_ nanoparticles increased syneresis in 28% and, MgO, SiO_2_ and Cr_2_O_3_ decreased syneresis behavior in 3%, 7.2% and 97.1%, respectively. Finally, for gels of 8000 mg∙L^−1^ of polymer, all nanoparticles had positive results. The sequence of syneresis percent reduction is Al_2_O_3_ < MgO < SiO_2_ < Cr_2_O_3_ with inhibitions of 6.25%, 43.7%, 53.8%, and 93.7% respectively.

From the displacement test, high additional oil recovery percent were obtained with the plugging of the Slim Tube with high permeability. Incremental oil recovery volumes of 55.9% and 45% were obtained for the low and medium permeability slim tubes, and only 1.9% of additional oil recovery was reached for the high permeability Slim Tube. This result indicates that the gel used was able to block the porous medium efficiently.

## Figures and Tables

**Table 1:** Mean particle size (D50), zeta potential, point of zero charges and, Brunauer–Emmett–Teller surface area (S_BET_) of Al_2_O_3_, MgO, Cr_2_O_3_, and SiO_2_ nanoparticles.

Material	D50 (nm)	Zeta Potential pH ~ 5	Point of Zero Charge	S_BET_ (m^2^/g)
Al_2_O_3_	35	43	9	43
MgO	80	18	11	21
Cr_2_O_3_	60	5	7	19
SiO_2_	11	−8	3	380

**Table 2:** Description of the gel strength based on Sydansk’s Code.

Gel Strength Code	Gel Description
1	Gel flows as polymer upon inversion.
2	Gel flows slightly slower than the polymer solution upon inversion.
3	Gel flows very slowly and does not fully leave the tube upon inversion.
4	When the bottle is inverted, the bubble barely makes it to the top of the tube.
5	When inverted, the bubble flows very slow and hardly makes it to the top of the bottle.
6	When inverted, the bubble does not make it to the top of the bottle
7	When inverted, the bubble makes it than a halfway to the top.
8	The bubble hardly moves off from the bottom of the tube.
9	When inverted, the gel surface is barely disturbed.
10	The gel surface remains flat.

**Table 3:** Sydansk’s code results for gel systems of acrylamide/sodium acrylate copolymer/chromium (III) Acetate at concentrations between 2000 and 8000 mg·L^−1^ in presence and absence of different nanoparticles at 70 °C ^1^ as a function of time.

	[Polymer]	2 h	4 h	8 h	24 h	48 h	1st Week	2nd Week	3rd Week	4th Week
**Base Gel**	**2000**	n	n	n	n	n	n	n	n	n
		1	1	1	1	1	1	1	1	1
								
	**4000**	n	n	s	s	s	s	s	s	s
		1	1	1	1	1	1	1	1	1
								
	**6000**	s	s	s	g	g	g	g	g	g
		1	1	2	4	4	6	6	6	6
								
	**8000**	s	s	s	g	g	g	g	g	g
		2	3	3	5	7	7	7	7	7
								
**Al_2_O_3_**	**2000**	n	n	n	n	n	n	n	n	n
		1	1	1	1	1	1	1	1	1
								
	**4000**	s	s	s	s	s	s	s	s	s
		1	2	3	3	3	3	3	3	3
								
	**6000**	s	s	g	g	g	g	g	g	g
		1	2	4	4	9	9	9	9	9
								
	**8000**	s	s	g	g	g	e	e	e	e
		2	2	3	5	8	10	10	10	10
								
**SiO_2_**	**2000**	n	n	n	n	n	n	n	n	n
		1	1	1	1	1	1	1	1	1
								
	**4000**	s	s	s	s	s	s	s	s	s
		1	2	3	3	3	3	3	3	3
								
	**6000**	s	s	g	g	g	g	g	g	g
		1	2	3	4	7	7	7	7	7
								
	**8000**	s	s	g	g	g	g	g	g	g
		2	2	3	5	9	9	9	9	9
								
**MgO**	**2000**	n	n	n	n	n	n	n	n	n
		1	1	1	1	1	1	1	1	1
								
	**4000**	s	s	s	s	s	s	s	s	s
		1	2	3	3	3	3	3	3	3
								
	**6000**	s	s	g	g	g	g	g	g	g
		1	3	4	4	8	8	8	8	8
								
	**8000**	s	s	g	g	g	e	e	e	e
		2	2	3	5	8	10	10	10	10
								
**Cr_2_O_3_**	**2000**	n	n	n	n	n	n	n	n	n
		1	1	1	1	1	1	1	1	1
								
	**4000**	n	n	s	s	s+	s+	s+	s+	s+
		1	1	2	2	2	2	2	2	2
								
	**6000**	s	s	s	g	g	g	g	g	g
		1	2	3	4	4	7	7	7	7
								
	**8000**	s	s	s	g	g	g	g	g	g
		2	2	2	5	8	8	8	8	8
								

Notes: ^1^ Color Scale.
12345678910

**Table 4:** Average conductivity of the porous medium before and after gel injection in the triple parallel Slim Tube test.

Conductivity (mL·min^−1^·KPa^−1^)	ST1 Low Permeability	ST2 High Permeability	ST3 Medium Permeability
Before	6.382	34.519	9.282
After	6.092	7.252 × 10^−2^	8.122
